# Abnormal expression of *PRKAG2-AS* results in dysfunction of cardiomyocytes through regulating *PRKAG2* transcription by interacting with *PPARG*

**DOI:** 10.1186/s13148-023-01591-w

**Published:** 2023-11-07

**Authors:** Xiao-Wei Song, Ting Su, Bo Li, Yun-Jie Huang, Wen-Xia He, Li-Li Jiang, Chang-Jin Li, Song-Qun Huang, Song-Hua Li, Zhi-Fu Guo, Hong Wu, Bi-Li Zhang

**Affiliations:** Department of Cardiology, Changhai Hospital, Second Military Medical University, 168 Changhai Road, Shanghai, 200433 China

**Keywords:** Heart failure, Myocardial infarction, Apoptosis, LncRNA, *PRKAG2*, PPARγ

## Abstract

The role of *PRKAG2* in the maintenance of heart function is well established, but little is known about how *PRKAG2 is* regulated in cardiomyocytes. In this study, we investigated the role of the lncRNA *PRKAG2-AS*, which is present at the *PRKAG2* promoter, in the regulation of *PRKAG2* expression. *PRKAG2-AS* expression was predominantly nuclear, as determined by RNA nucleoplasmic separation and fluorescence in situ hybridization. Knockdown of *PRKAG2-AS* in the nucleus, but not the cytoplasm, significantly decreased the expression of *PRKAG2b* and *PRKAG2d*. Interestingly, we found that *PRKAG2-AS* and its target genes, *PRKAG2b* and *PRKAG2d*, were reduced in the hearts of patients with ischemic cardiomyopathy, suggesting a potential role for *PRKAG2-AS* in myocardial ischemia. Indeed, knockdown of *PRKAG2-AS* in the nucleus resulted in apoptosis of cardiomyocytes. We further elucidated the mechanism by which *PRKAG2-AS* regulates *PRKAG2* transcription by identifying 58 *PRKAG2-AS* interacting proteins. Among them, *PPARG* was selected for further investigation based on its correlation and potential interaction with *PRKAG2-AS* in regulating transcription. Overexpression of *PPARG*, or its activation with rosiglitazone, led to a significant increase in the expression of *PRKAG2b* and *PRKAG2d* in cardiomyocytes, which could be attenuated by *PRKAG2-AS* knockdown. This finding suggests that *PRKAG2-AS* mediates, at least partially, the protective effects of rosiglitazone on hypoxia-induced apoptosis. However, given the risk of rosiglitazone in heart failure, we also examined the involvement of *PRKAG2-AS* in this condition and found that *PRKAG2-AS*, as well as *PRKAG2b* and *PRKAG2d*, was elevated in hearts with dilated cardiomyopathy (DCM) and that overexpression of *PRKAG2-AS* led to a significant increase in *PRKAG2b* and *PRKAG2d* expression, indicating that up-regulation of *PRKAG2-AS* may contribute to the mechanism of heart failure by promoting transcription of *PRKAG2*. Consequently, proper expression of *PRKAG2-AS* is essential for maintaining cardiomyocyte function, and aberrant *PRKAG2-AS* expression induced by hypoxia or other stimuli may cause cardiac dysfunction.

## Introduction

The primary cause of death worldwide, heart failure, is directly correlated with the energy metabolism of cardiomyocytes. Adenosine 5'-monophosphate (AMP)-activated protein kinase (AMPK) plays a critical role in regulating myocardial energy balance in the myocardium [1]. AMPK functions as a cellular energy sensor that modulates ATP production by increasing glucose uptake and fatty acid oxidation and inhibiting ATP consumption, such as protein and lipid synthesis, under various cellular stresses [2]. AMPK is a heterotrimeric complex consisting of a catalytic subunit α (PRKAA), a regulatory subunit β (PRKAB), and a regulatory subunit γ (PRKAG). Mutations in the *PRKAG2* gene, which encodes the γ2 regulatory subunit highly expressed in the heart, lead to multiple diseases due to abnormal AMPK activity [3]. However, the regulatory mechanism for *PRKAG2* expression in cardiomyocytes remains unclear.

The *PRKAG2* gene, which has 16 exons and codes for 569 amino acids, is located on chromosome 7q36. Mutations in *PRKAG2* result in various diseases due to abnormal AMPK activity [4]. We reported that a family was affected with *PRKAG2* syndrome due to the G100S mutation of *PRKAG2* [5]. The key pathological changes observed in this family included myocardial hypertrophy, ventricular pre-excitation, and progressive conduction system abnormalities. Studies investigating mutations in different sites of the *PRKAG2* gene have revealed that *PRKAG2* plays a crucial role in maintaining heart function. Therefore, exploring the mechanisms that regulate PRKAG2 gene expression in cardiomyocytes is crucial. Numerous processes, including epigenetics [6–8], RNA splicing [9–11], RNA capping and polyadenosine [12], RNA modification [13], translocalization [14], mRNA translation and RNA decay [15, 16], can regulate gene expression. Recent evidence indicates noncoding RNAs are crucial in regulating gene expression [17]. Hence, we aim to investigate whether the regulation of *PRKAG2* gene expression could be mediated through noncoding RNAs. Long noncoding RNAs (lncRNAs) are ncRNAs longer than 200 bases and account for 4% to 9% of mammalian transcripts [18]. Several studies have suggested that lncRNAs are important transcriptional regulators and are involved in regulating the development of various cardiovascular disorders. By directly affecting the transcription of nearby genes or by spreading from their transcription site to act on distant genes or other chromosomes, LncRNAs can regulate gene expression in both cis- and trans-modes [19].

This study aims to determine whether lncRNAs are involved in regulating *PRKAG2* expression as well as their functions and mechanisms in cardiovascular disease. Specifically, we investigated the *PRKAG2* locus and identified a lncRNA called *PRKAG2-AS*, which is the antisense RNA of *PRKAG2* and is commonly expressed in human tissues. The function and mechanism of *PRKAG2-AS* in the cardiovascular system remain unclear. Given that *PRKAG2* is the AMPK regulatory subunit, we hypothesize that *PRKAG2-AS* regulates cardiomyopathy through the transcriptional control of *PRKAG2*. As a result, our study found that *PRKAG2-AS* can act as a regulatory element to modulate AMPK activity by regulating the transcription of the *PRKAG2* gene and participate in regulating the occurrence of cardiovascular disease.

## Materials and methods

### Human heart sample collection

The collection of clinical specimens was approved by the Ethics Committee of the Second Military Medical University (approval code: NMU8217021385). As described before [20, 21], all human heart samples used in this study were from patients who had organ transplants in ChangHai Hospital from 2012 to 2018 with informed consent from the patients or their families (approval code: SMMU913293039). ICM and DCM samples came from the abandoned hearts of heart transplant acceptors after surgery. Normal heart samples were obtained from donors whose livers or other organs were transplanted, while the hearts were unsuitable for transplantation. Fresh specimens were obtained during clinical surgery and stored at -80 °C after freezing with liquid nitrogen.

### Cell culture and transfection with siRNAs or oligos

Cells were incubated in DMEM complete medium containing 10% fetal bovine serum and 1% biclonal antibody at 37 °C. siRNAs or oligos were commercially obtained from GenePharma Corp. (Suzhou, China). Following the manufacturer's instructions, 20 nM diluted siRNAs or oligos were used to transfect cells (5 µl/well) using Lipofectamine 2000. Nontargeted siRNA or oligos were used as negative controls (NCs).

### Establishment and transfection of adenovirus

The adenovirus serotype 5 (Ad5) system was employed to overexpress *PRKAG2-AS*, *PRKAG2*, and *PPARG* in cardiomyocytes. Briefly, the sequences of these genes were cloned onto pAdtrack vectors (adenoviral shuttle vector) and transferred into BJ5183 bacteria to recombine with pAdEasy. *AdEasy-PRKAG2-AS-Cys4* Binding sequence, *AdEasy-PRKAG2,* and *AdEasy-PPARG* were digested with *Pac I* to expose its inverted terminal repeats (ITRs) and then transfected into 293A cells to construct Adenovirus. Following amplification and titer determination, cardiomyocytes were typically transfected using 50 Multiplicity of Infection (MOI) to overexpress these genes. Forty-eight hours after transfection, the cardiomyocytes were harvested for further analysis.

### Hypoxic model and hypertrophic model of cardiomyocytes

AC16 cells were seeded and transferred to a low-oxygen incubator for a 24-h exposure to 1% O_2_-94% N_2_-5% CO_2_. Primary cardiomyocytes were isolated from the hearts of 3-day-old SD rats. The hearts were removed and washed in a pre-cooled Hank's balanced salt solution, cut into tissue blocks of approximately 1 mm^3^, and then digested with collagenase I overnight at 4 °C. The next day, cardiomyocytes were collected by centrifugation at 1500 rpm for 10 min and cultured in DMEM complete medium containing 10% fetal bovine serum and 1% penicillin–streptomycin. After pre-seeding for 2 h to remove fibroblasts, cardiomyocytes were seeded in 6-well plates and cultured in a 37 °C, 5% CO_2_ incubator. Thirty-six hours later, the DMEM medium was replaced with medium containing 0.1 mM BrdU. After another 24 h, the medium was changed with serum-free DMEM medium containing Brdu and cultured for another 24 h before 100 uM phenylephrine was added to stimulate cardiomyocyte hypertrophy.

### Apoptosis detection using flow cytometry

The rate of cell death was determined by an Annexin V-FITC Apoptosis Detection Kit (Beyotime, C1062) according to the manufacturer's instructions. Cardiomyocytes were digested with trypsin, washed, double-stained with AV and PI, and then analyzed using the previously described methods with a flow cytometer.

### Separation of cardiomyocyte nuclear and cytoplasmic fractions

According to the manufacturer's instructions, the nuclear and cytoplasmic fractions from cardiomyocytes were separated with a PARIS Kit (Life Technologies, AM1921). Briefly, AC16 cells were cultured in 10-cm dishes and collected with 0.25% trypsin. The cells were resuspended with 250 ul Cell Disruption Buffer, lysed with 250 ul Cell Fractionation Buffer for 5 min, and then subjected to centrifugation at 500 g, 4 °C for 3 min. The supernatant was taken for further extraction of cytoplasmic RNA. The pellet was centrifuged again after being washed with 250 ul Cell Fractionation Buffer. The remaining pellet was used to extract nuclear RNA with Trizol (Invitrogen, China, Shanghai).

### Quantitative reverse transcriptase-polymerase chain reaction

Total RNA was extracted with TRIzol (Invitrogen, China, Shanghai). Reverse transcription was performed with M-MLV using random primers. The amounts of target genes were determined by qPCR using SYBR Green methods with a LightCycler-480 machine. The levels of *GAPDH* were used as endogenous controls with the 2^ΔΔ Ct^ analysis method.

### Western blot

Samples were treated with ultrasound on ice after being lysed with RIPA buffer (Beyotime, Hangzhou, China). After boiling for 10 min, a total of 40 μg protein/well was electrophoresed in a 10% SDS-PAGE gel and transferred to PVDF membrane (Bio-Rad, America). The membranes were blocked with 5% skim milk for 2 h and then incubated overnight with primary antibodies and HRP-conjugated secondary antibodies for 2 h. The protein levels were detected by ECL (XinSaiMei, Suzhou, China). Anti-*PRKAG2* (CST, #2536), anti-*PPARG* (A19676, Abclonal, Wuhan, China), and anti-*GAPDH* rabbit mAb (ABways, Ab0037) were the antibodies that were used.

### RNP immunoprecipitation (RIP)

AC16 cells were cultured in T75 flasks and transfected for 24 h with Ad-GFP or Ad-Cys4 + Ad-*PRKAG2-AS* fusing with Cys4 binding sites [22, 23] at 50 MOI. Cells from 4 flasks for each treatment were collected and crosslinked with 1% glutaraldehyde. The cross-linking reaction was stopped with glycine solution. The cells were collected and lysed with 2 ml lysis buffer (50 mM Tris–Cl, 1% NP-40, 0.5% sodium deoxycholate, 0.05% SDS, 1 mM EDTA, 150 mM NaCl, RNase inhibitor, and protease inhibitors) and then sonicated on ice for 15 min. Insoluble material was removed by centrifugation. The supernatants were mixed with protein A/G-beads precoated with the Flag-Antibody (20 ul) for 2 h at 4 °C, followed by extensive washing with RIPA-Buffer containing protease and RNase inhibitors. The beads containing the immunoprecipitated samples were collected and resuspended in solution buffer (50 mM Tris–Cl, 5 mM EDTA, 10 mM DTT, and 1% SDS) and incubated at 70 °C for 45 min to reverse the crosslinks. The RNA was extracted with Trizol, and proteins were saved for analysis.

### Pulling down *PRKAG2-AS* binding proteins with biotin-oligo

AC16 cells from 10 T75 flasks were collected and cross-linked with 1% glutaraldehyde, as indicated above. Following lysis, the supernatants were split into two groups: one for hybridization with control oligos and the other for *PRKAG2-AS* targeting oligo probes. *PRKAG2-AS* binding proteins were isolated with DynaMag-2 magnetic beads. After washing three times, the beads were resuspended in a solution buffer, and the proteins were collected for further analysis. Shanghai AiFu Biotechnology Company provided Mass Spectrometry as a technique service.

### Statistical method

The data were statistically analyzed using SigmaPlot software. The results are expressed as mean ± standard deviation. T-test was used to analyze the difference between the experimental and control groups, and *p* < 0.05 was considered statistically significant.

## Results

### LncRNA *PRKAG2-AS* is located on the promoter region of *PRKAG2*

**Fig. 1:**
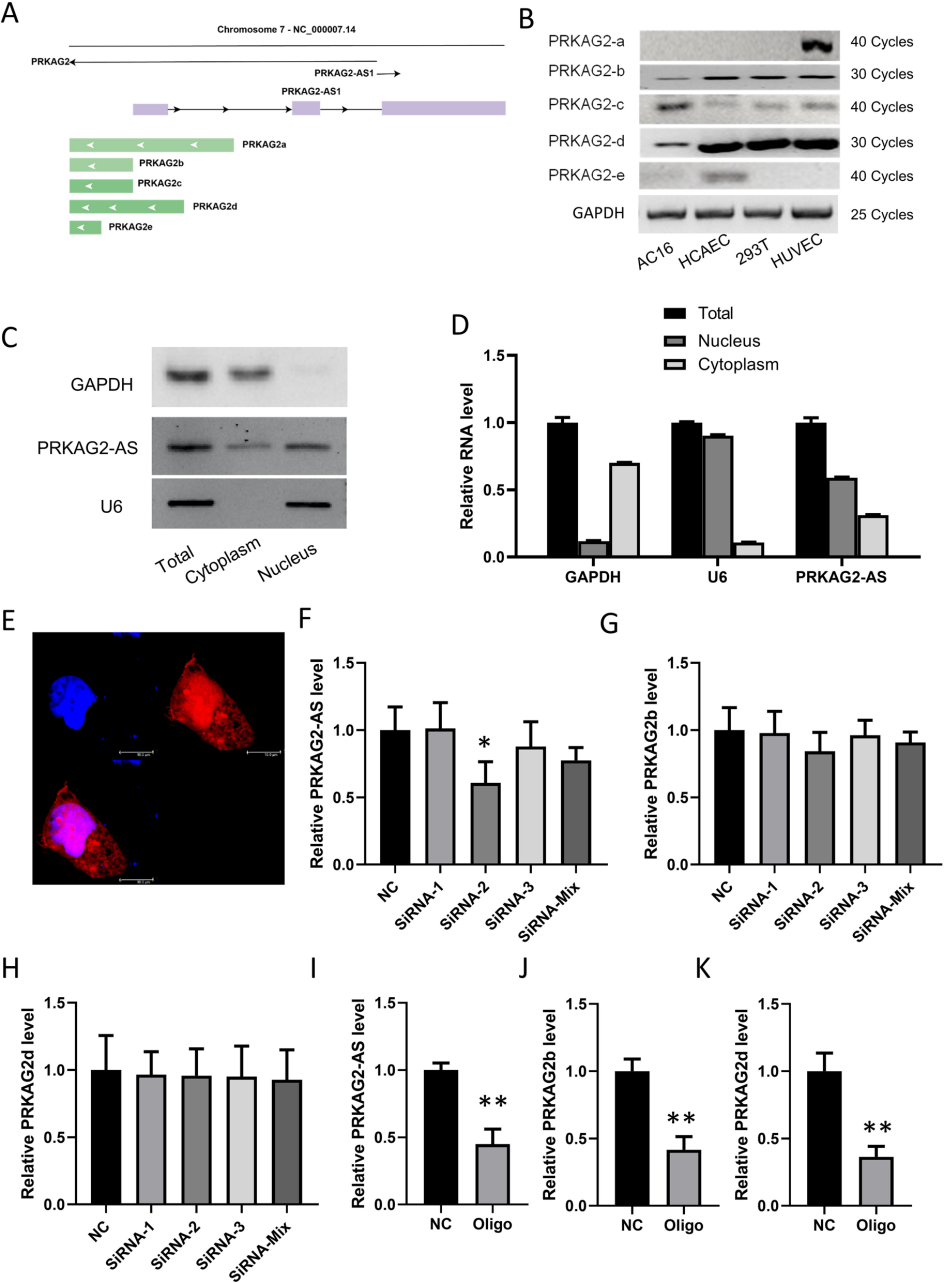
*PRKAG2b* and *PRKAG2d* were transcriptionally regulated by nuclear *PRKAG2-AS.* **A** A long noncoding RNA called *PRKAG2-AS* is found within the promoter region of the *PRKAG2* gene, which has five isoforms resulting from various transcription starting sites. **B** RT-PCR analysis of the expression profiles of the 5 *PRKAG2* isoforms in AC16, HCAEC, 293 T, and HUVEC cells. **C**-**D** RNA nucleoplasmic separation of AC16 cells and qRT-PCR revealed the nuclear localization of *PRKAG2-AS*. *U6* and *GAPDH* were used as controls for nuclear and cytoplasmic localization, respectively. **E** Fluorescence in situ hybridization using 3 biotin-oligos targeting *PRKAG2-AS* showed its predominant localization within the nucleus. **F** Knockdown of *PRKAG2-AS* in AC16 cells by siRNA. **G**-**H** The expression of *PRKAG2b* and *PRKAG2d* was unaffected by the siRNA-mediated knockdown of *PRKAG2-AS.* (I) *PRKAG2-AS* was knocked down in the nucleus by antisense oligonucleotides. **J**-**K** The mRNA levels of *PRKAG2b* and *PRKAG2d* were significantly reduced in cells subjected to knockdown of nuclear *PRKAG2-AS* by antisense oligonucleotides

Five *PRKAG2* isoforms that originate from different transcription starting sites were described on the NCBI website (Fig. 1A). The expression profiles of *PRKAG2* isoforms were analyzed by RT-PCR in AC16, HCAEC, 293 T, and HUVEC cells using *GAPDH* as the internal control. *PRKAG2a* and *PRKAG2e* were hard to be detected in AC16 cells. Although the expression of *PRKAG2c* was predominant in AC16 than in other cell lines, it needed 10 more replication cycles to be detected than *PRKAG2b* and *PRKAG2d* in the semi-quantitative RT-PCR. *PRKAG2b* and *PRKAG2d* had relatively higher expression in AC16 cardiomyocytes (Fig. 1B). On the promoter regions of *PRKAG2*, there is a LncRNA called *PRKAG2-AS.* To determine the localization of *PRKAG2-AS* in cardiomyocytes, RNA nucleoplasmic isolation of AC16 cells and qRT-PCR was performed with *U6* and *GAPDH* as positive controls for RNA located in the nucleus and cytoplasm, respectively (Fig. 1C–D), showing that *PRKAG2-AS* was mainly expressed in the nucleus (Fig. 1C–D). Further, fluorescence in situ hybridization was performed using three biotin-oligos targeting *PRKAG2-AS*. Results showed that *PRKAG2-AS* was present inside and outside the nucleus, but mainly within the nucleus (Fig. 1E).

After cytoplasmic *PRKAG2-AS* was knocked down using siRNAs in AC16 cells (Fig. 1F), the expression of *PRKAG2b* and *PRKAG2d* was detected, showing that alteration of *PRKAG2-AS* by siRNA did not affect the expression of *PRKAG2b* and *PRKAG2d* (Fig. 1G–H). This might occur as a result of the major location of *PRKAG2-AS* being in the nucleus, where RNA-induced Silencing Complex might not be able to reach. Antisense oligos with the same sequence as siRNA-2 were used to knock down *PRKAG2-AS* in the nucleus (Fig. 1I). As a result, *PRKAG2b* and *PRKAG2d* mRNA expression levels were significantly down-regulated by oligo targeting *PRKAG2-AS* (Fig. 1J–K). It can be concluded that nuclear *PRKAG2-AS*, but not cytoplasmic *PRKAG2-AS*, plays a crucial role in regulating the transcription of *PRKAG2b* and *PRKAG2d* in AC16 cells.

### Positive correlation between *PRKAG2-AS* and *PRKAG2* in cardiac ischemia

**Fig. 2:**
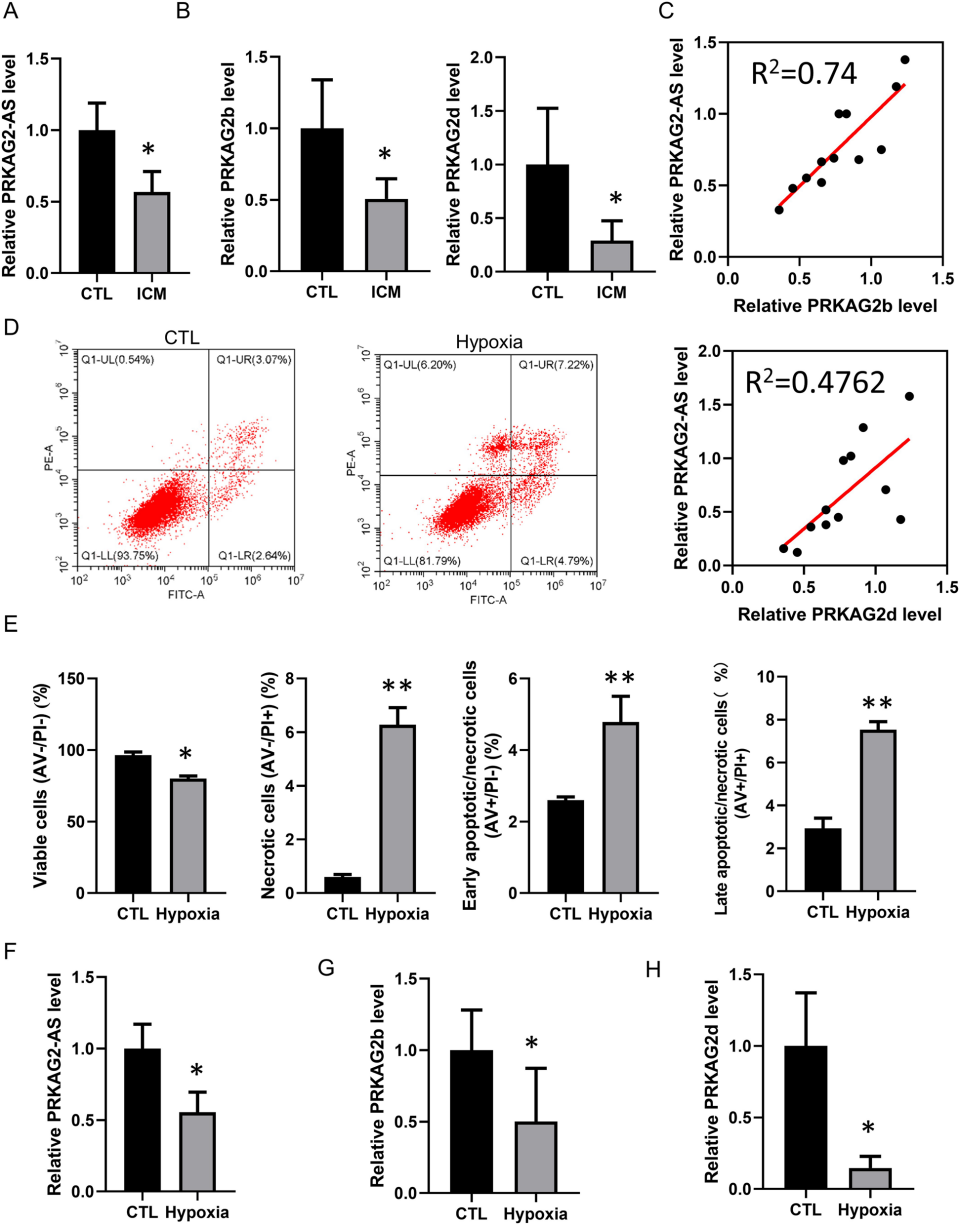
A positive correlation between *PRKAG2-AS* and *PRKAG2* in cardiac ischemia. **A** An analysis of 6 heart samples from healthy donors and 6 heart samples from patients with ischemic cardiomyopathy showed a significant reduction in the expression of *PRKAG2-AS* in the hearts of those suffering from ischemic cardiomyopathy than in the hearts of healthy individuals. **B** Expression of *PRKAG2b* and *PRKAG2d* was reduced in human ischemic hearts. **C** A positive correlation between the expression of *PRKAG2-AS* and *PRKAG2b* or *PRKAG2d* was observed in ischemic cardiomyopathy. **D**-**E** Flow cytometry analysis demonstrated apoptosis and necrosis of cardiomyocytes induced by hypoxia. **F**–**H** The expression of *PRKAG2-AS*, *PRKAG2b*, and *PRKAG2d* was significantly reduced in hypoxic cardiomyocytes

To investigate the potential involvement of *PRKAG2-AS* in ischemic cardiomyopathy, we detected the expression of *PRKAG2-AS* in six heart samples from healthy donors and six heart samples from patients with ischemic cardiomyopathy. The results showed that *PRKAG2-AS* is significantly reduced in the hearts of patients with ischemic cardiomyopathy compared to healthy individuals (Fig. 2A). Notably, the expression of *PRKAG2b* and *PRKAG2d* in human ischemic hearts was also decreased (Fig. 2B, C). A positive correlation between the expressions of *PRKAG2-AS* and *PRKAG2b* or *PRKAG2d* was found after additional investigation (Fig. 2C). Therefore, we established a cardiomyocyte hypoxic model by exposing AC16 cells to 1% O_2_-94% N_2_-5% CO_2_ for 24 h. Flow cytometry data demonstrated that hypoxia-induced apoptosis and necrosis of cardiomyocytes (Fig. 2D–E). After confirming the success of the hypoxic model, we measured the expression levels of *PRKAG2-AS*, *PRKAG2b*, and *PRKAG2d* in hypoxic cardiomyocytes using qRT-PCR. A significant reduction in the expression of *PRKAG2-AS*, *PRKAG2b*, and *PRKAG2d* was observed in hypoxic cardiomyocytes (Fig. 2F–H). These findings suggest that the down-regulation of *PRKAG2-AS* in cardiac ischemia may play crucial roles in cardiomyocyte apoptosis or necrosis by suppressing the expression of *PRKAG2b* and *PRKAG2d*.

### Knockdown of *PRKAG2-AS-induced* apoptosis of cardiomyocytes

**Fig. 3:**
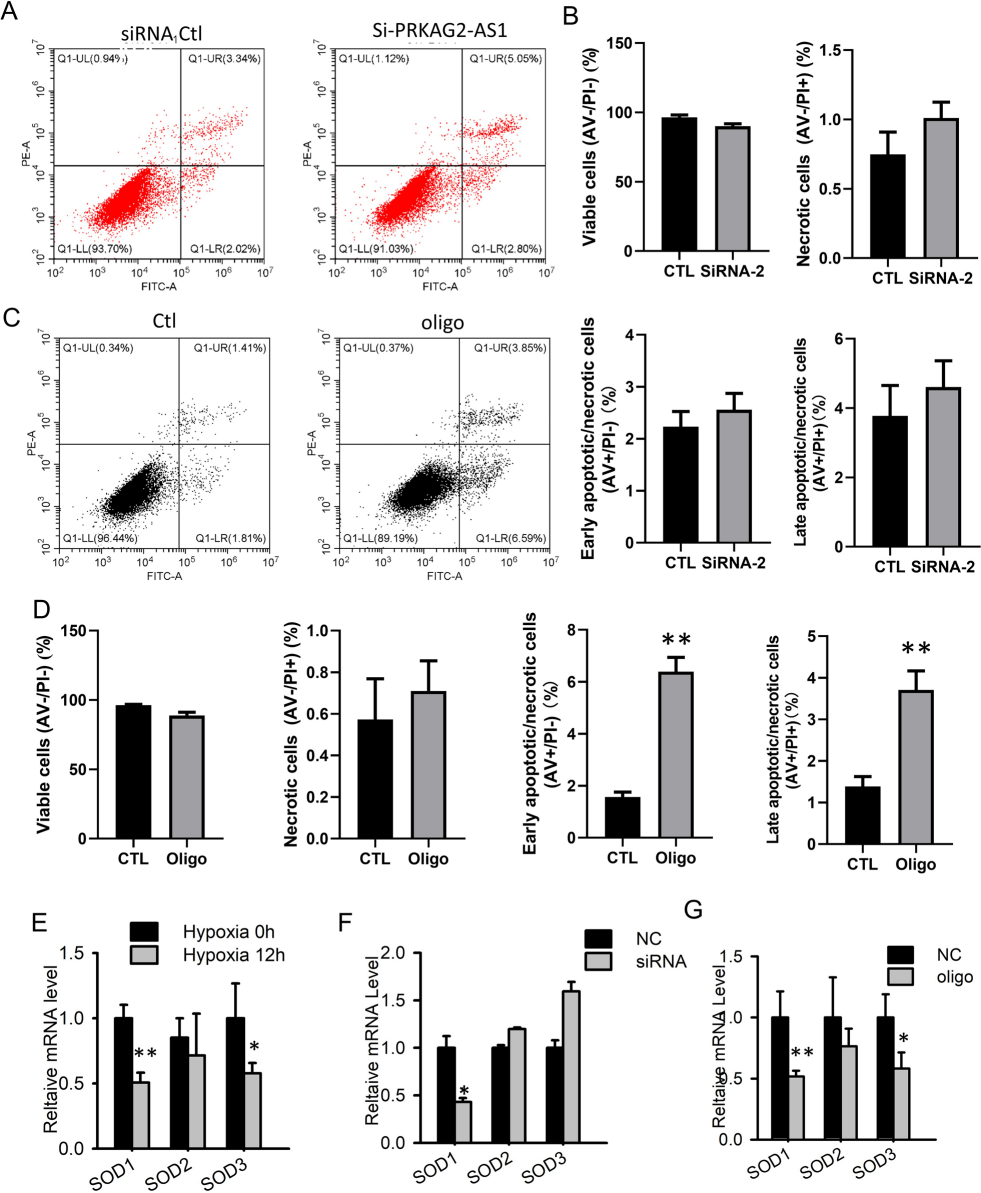
Knockdown of *PRKAG2-AS*-induced apoptosis of cardiomyocytes. **A**-**B** Apoptosis or necrosis of cardiomyocytes was not significantly increased after cytoplasmic *PRKAG2-AS* was knocked down by siRNA compared to NC controls. **C**-**D** Knockdown of nuclear *PRKAG2-AS* by antisense oligos led to a significant increase in apoptosis of cardiomyocytes. **E** Analysis of *SOD1*, *SOD2,* and *SOD3* expression in the hypoxic cardiomyocyte model revealed that *SOD1* and *SOD3* were down-regulated by hypoxia. **F** Cytoplasmic knockdown of *PRKAG2-AS* by siRNA decreased the expression level of *SOD1* but did not affect the expression of *SOD2* and *SOD3*. **G** Nuclear knockdown of *PRKAG2-AS* reduced the expression of *SOD1* and *SOD3* in cardiomyocytes

To gain insight into the involvement of *PRKAG2-AS* in cardiomyocyte hypoxia, we used siRNA to knock down cytoplasmic *PRKAG2-AS* and assessed the effect on cardiomyocyte apoptosis and necrosis using flow cytometry. The results showed that knocking down cytoplasmic *PRKAG2-AS* had no significant effect on apoptosis or necrosis of cardiomyocytes compared to the negative control (Fig. 3A–B). Subsequently, we knocked down nuclear *PRKAG2-AS* with antisense oligos, which significantly increased cardiomyocyte apoptosis (Fig. 3C–D). Superoxide dismutase (SOD), among other antioxidant enzyme systems, has been shown in various clinical and experimental studies to play a critical role in maintaining intracellular reactive oxygen species (ROS) homeostasis in heart failure tissues [24]. SOD is considered the first line of defense against the accumulation of free radicals, and it can be categorized into three types based on different metal cofactors: copper-zinc superoxide dismutase (*SOD1*), manganese superoxide dismutase (MnSOD/*SOD2*), and extracellular copper-zinc superoxide dismutase (*SOD3*) [24]. Here, we assessed the expression of *SOD1*, *SOD2*, and *SOD3* in the cardiomyocyte hypoxia model and found down-regulation of *SOD1* and *SOD3* (Fig. 3E). Interestingly, cytoplasmic knockdown of *PRKAG2-AS* by siRNA led to a decrease in the expression level of *SOD1* but had no significant effect on the expression of *SOD2* and *SOD3* (Fig. 3F). In cardiomyocytes subjected to nuclear *PRKAG2-AS* knockdown, the expression patterns of *SOD1*, *SOD2*, and *SOD3* were identical with those observed in hypoxia-treated cardiomyocytes, demonstrating a reduction in *SOD1* and *SOD3* expression and no effect on *SOD2* (Fig. 3G).

### Knockdown of *PRKAG2b* and *PRKAG2d* resulted in apoptosis of cardiomyocytes

**Fig. 4:**
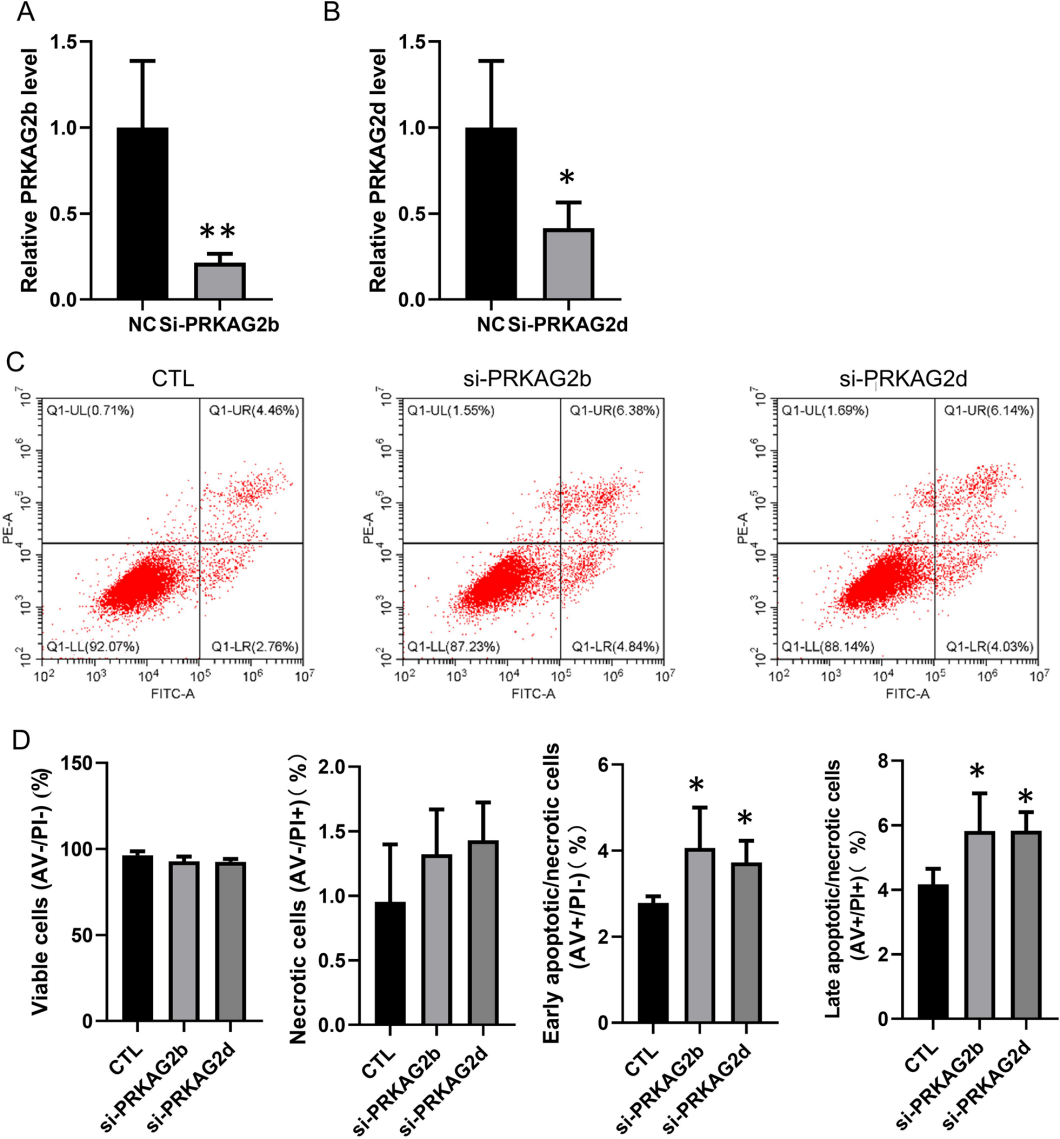
Knockdown of *PRKAG2b* and *PRKAG2d* resulted in apoptosis of cardiomyocytes. **A**-**B** The expression of *PRKAG2b* and *PRKAG2d* was significantly reduced by siRNAs. **C**-**D** Knocking down *PRKAG2b* and *PRKAG2d* resulted in a slight increase in apoptosis of cardiomyocytes

As demonstrated above, *PRKAG2-AS* plays a significant role in positively regulating the expression of *PRKAG2b* and *PRKAG2d*. Furthermore, we observed a down-regulation of *PRKAG2-AS* in cardiac ischemia and discovered that knockdown of *PRKAG2-AS* leads to apoptosis. Based on these findings, we proposed that reducing the expression of *PRKAG2b* and *PRKAG2d* might underlie the regulatory effect of *PRKAG2-AS* knockdown on apoptosis. To test this hypothesis, we employed siRNA to knock down *PRKAG2b* and *PRKAG2d* and assessed apoptosis using flow cytometry. Our data demonstrate that the siRNA effectively reduced the expression of *PRKAG2b* and *PRKAG2d*, as verified by qRT-PCR (Fig. 4A–B). Moreover, we noted a slight increase in cardiomyocyte apoptosis upon knocking down *PRKAG2b* and *PRKAG2d* (Fig. 4C–D). These results suggest that the down-regulation of *PRKAG2b* and *PRKAG2d* in myocardial ischemia contributes to hypoxia-induced apoptosis of cardiomyocytes.

### PPARγ plays an essential role in *PRKAG2-AS* regulating *PRKAG2* transcription

**Fig. 5:**
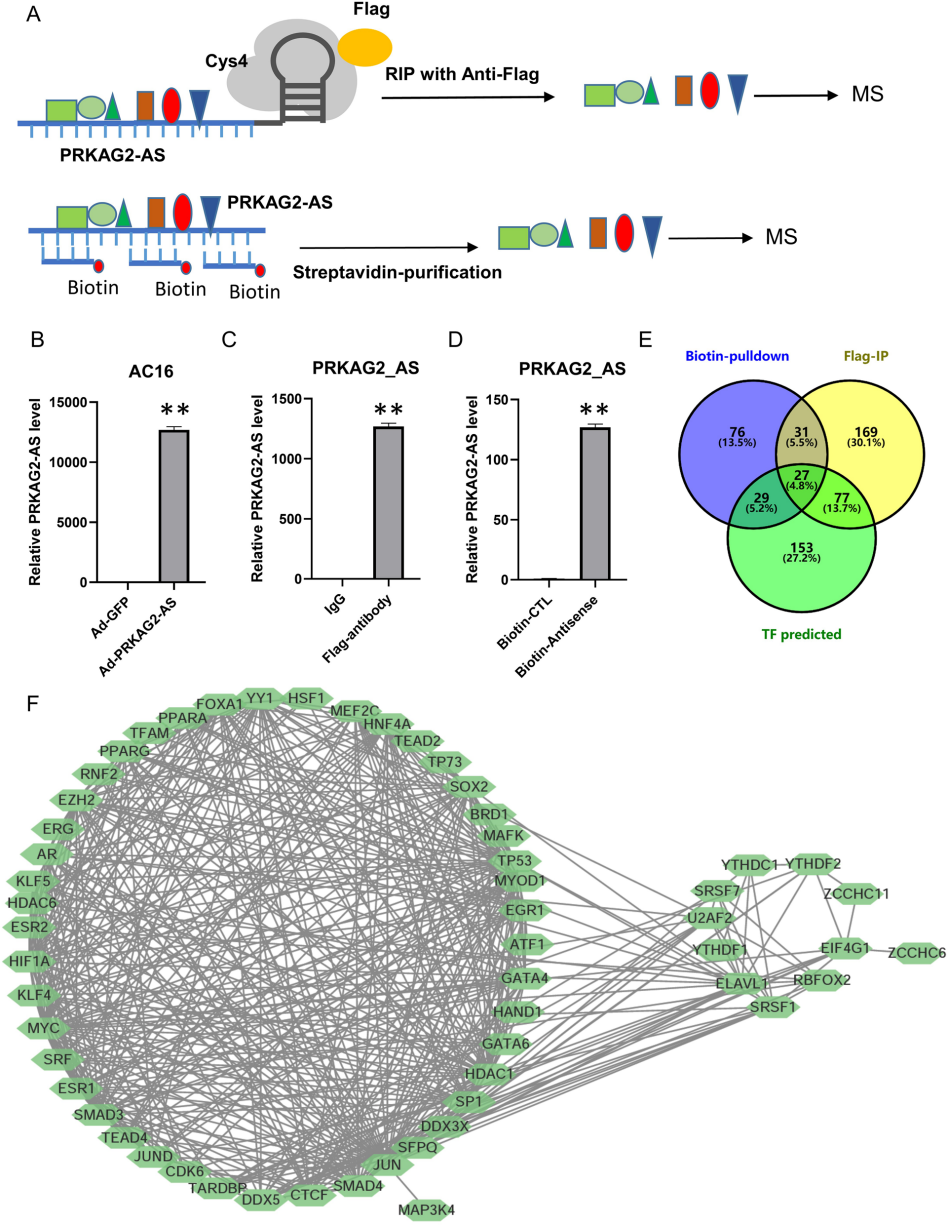
*PRKAG2-AS* interacting proteins. **A** Two strategies were employed to screen for *PRKAG2-AS* interacting proteins. **B** The expression of *PRKAG2-AS* by adenovirus was confirmed by qRT-PCR. **C** Successful RIP was confirmed by the result that Flag antibody could enrich *PRKAG2-AS* relative to the IgG control. **D** Biotin-labeled antisense oligos were able to purify *PRKAG2-AS*. **E** RIP and biotin-antisense purification identified 304 and 162 proteins, respectively, with 58 proteins appearing in both sets. **F** The functional relationships between the 58 identified *PRKAG2-AS* binding proteins were analyzed through STRING and Cytoscape

We hypothesized that *PRKAG2-AS* might directly interact with transcription factors to regulate the expression of *PRKAG2b* and *PRKAG2d.* We employed two strategies to identify *PRKAG2-AS* interacting proteins in cardiomyocytes (Fig. 5A). First, we established a *PRKAG2-AS*-overexpressing adenovirus containing a Csy4 binding site. Following the transfection of Ad-*PRKAG2-AS* and Ad-Cys4-Flag into AC16 cells for 24 h, the expression of *PRKAG2-AS* was confirmed by qRT-PCR (Fig. 5B). We subsequently purified *PRKAG2-AS* interacting proteins through RNP immunoprecipitation with a Flag antibody and compared it with an IgG control. The successful enrichment of *PRKAG2-AS* by Flag antibody indicates that the RIP process worked as intended. The other method is purifying biotin-labeled antisense oligos paired with *PRKAG2-AS* in AC16 lysis using streptavidin beads (Fig. 5A). We also verified that *PRKAG2-AS* was enriched through magnetic bead purification (Fig. 5D). Through mass spectrometry analysis, we identified 304 and 162 proteins using RIP and biotin-antisense purification, respectively (Fig. 5E). Among them, 58 proteins were identified by both methods. Using the TF-Mapper tool [25] (www.tfmapper.org), we predicted that 27 of the *PRKAG2-AS* binding proteins might bind to the promoter region of *PRKAG2* (Fig. 5E). We performed protein interaction analysis through the STRING database for the 58 *PRKAG2* binding proteins, and the interaction between these proteins is illustrated in Fig. 5F. This network included a collection of transcription factors, such as HIF1a, SMAD2, SMAD3, SMAD4, PPARG, PPARA, MEF2C, and SFPQ, along with several RNA-binding proteins, including SRSF1, SRSF7, U2AF2, ELAVL1, RBFOX2, YTHDF1, YTHDF2, and YTHDC1 (Fig. 5F).

**Fig. 6:**
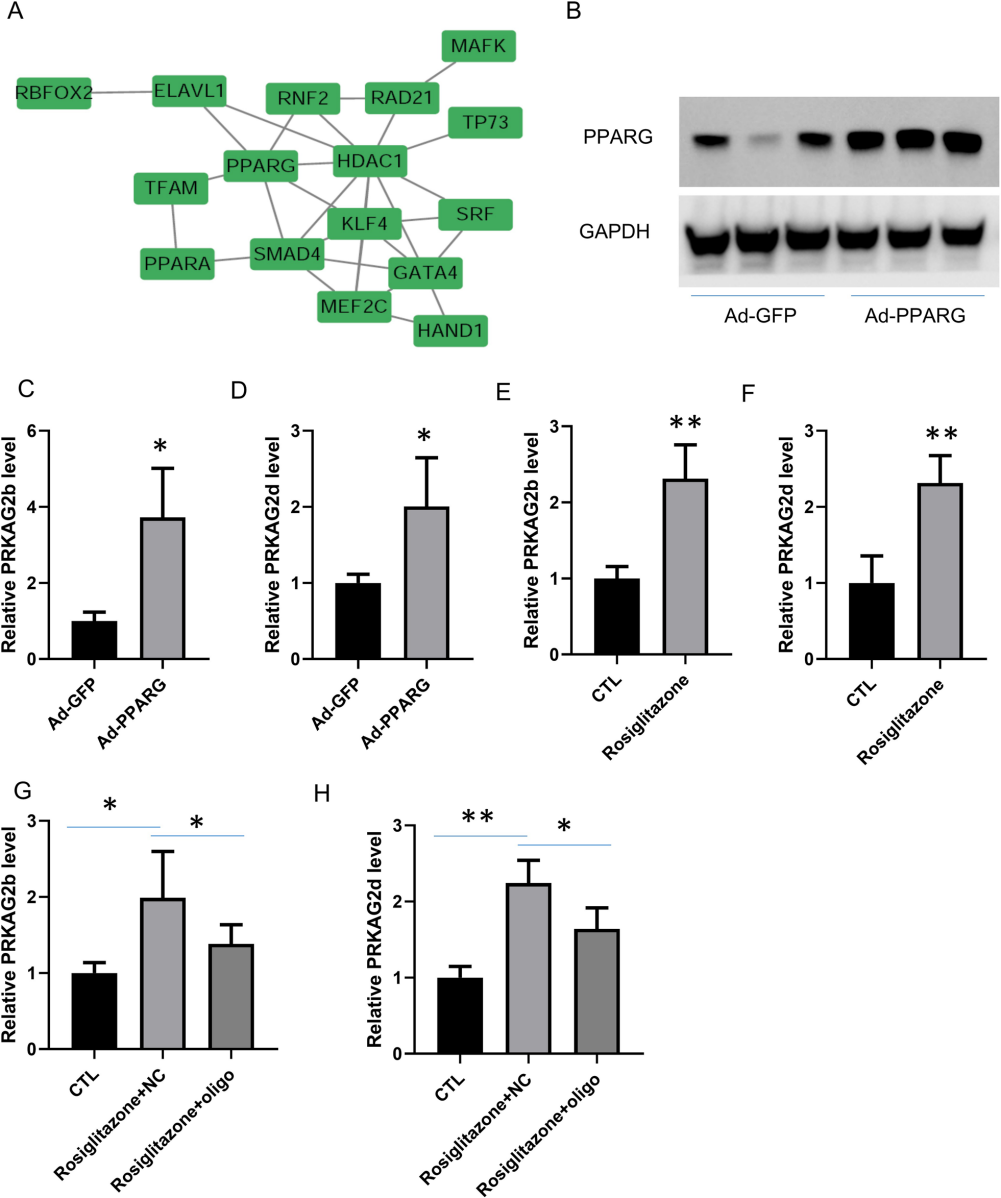
*PRKAG2-AS* regulated *PRKAG2b* and *PRKAG2d* by directly interacting with PPARγ. **A** The top ten positive relative proteins between *PRKAG2* and these 58 proteins in the GSE57338 and GSE79962 datasets were used to construct a network by STRING and Cytoscape, in which *PPARG* and HDAC1 occupied a pivotal position. **B**–**D** Overexpression of *PPARG* promoted the transcription of *PRKAG2b* and *PRKAG2d* in AC16 cells. **E**–**F** The PPARγ agonist rosiglitazone significantly up-regulated the expression of *PRKAG2b* and *PRKAG2d* in cardiomyocytes. **G**-**H** The effects of rosiglitazone on *PRKAG2b* and *PRKAG2d* transcription were mediated by *PRKAG2-AS*

To identify the factors involved in *PRKAG2-AS* regulation of *PRKAG2* expression in cardiomyocytes, we calculated the correlation of mRNA expression between *PRKAG2* and the 58 proteins identified by mass spectrometry in GSE57338 [26] and GSE79962 [27] datasets. The top ten positively correlated proteins in each GSE dataset were collected and analyzed using STRING websites and Cytoscape, revealing that *PPARG* and HDAC1 occupy central positions in the network (Fig. 6A). HDAC1 typically negatively regulates, and *PPARG* often positively regulates the transcription of their binding genes. Consequently, we focused on the effects of *PPARG* on *PRKAG2-AS*-mediated transcriptional regulation of *PRKAG2*. We overexpressed *PPARG* in AC16 cells (Fig. 6B) and monitored the expression of *PRKAG2b* and *PRKAG2d*, demonstrating that PPARγ plays a pivotal role in the transcriptional regulation of *PRKAG2* (Fig. 6C–D). We also tested the effect of rosiglitazone, a clinically used PPARγ agonist, on the expression of *PRKAG2b* and *PRKAG2d* in cardiomyocytes (Fig. 6E–F), showing that rosiglitazone (0.3 μM) significantly increased the expression of *PRKAG2b* and *PRKAG2d*. To investigate whether the effects of rosiglitazone on regulating the transcription of *PRKAG2* are mediated by *PRKAG2-AS*, we knocked down nuclear *PRKAG2-AS* and detected the expression of *PRKAG2b* and *PRKAG2d*. The results showed that up-regulation of *PRKAG2b* and *PRKAG2d* induced by rosiglitazone can be attenuated by *PRKAG2-AS* knockdown (Fig. 6G–H). Our findings indicated that the protective effects of rosiglitazone on hypoxia-induced apoptosis were, at least partially, through *PRKAG2-AS*.

### Up-regulation of *PRKAG2-AS* might result in abnormal function of hearts

**Fig. 7:**
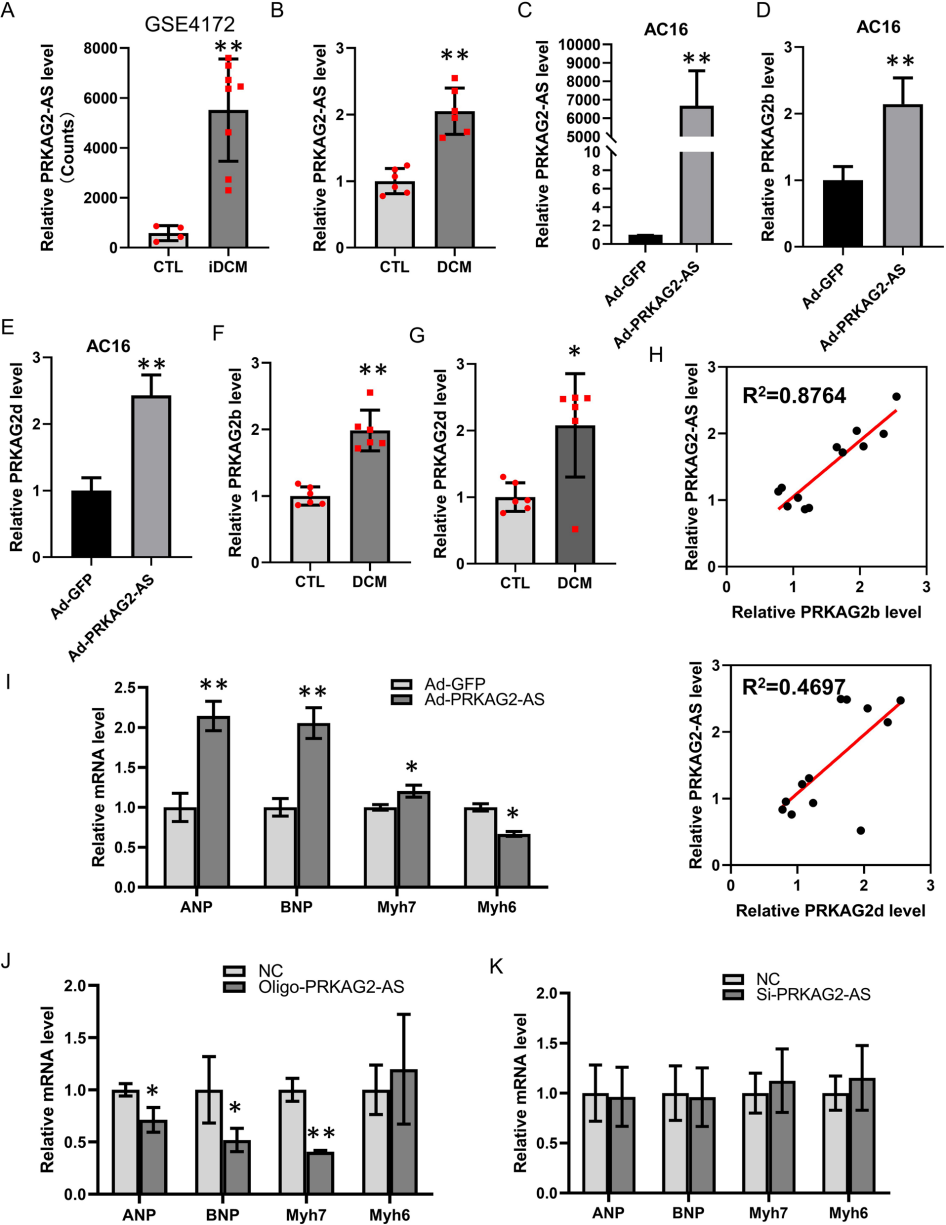
Up-regulation of *PRKAG2-AS* may result in abnormal heart function. **A** Analysis of the GSE4172 dataset showed elevated expression of *PRKAG2-AS* in the myocardium of patients with inflammatory dilated heart failure. **B** *PRKAG2-AS* expression was significantly up-regulated in DCM heart samples collected for this study. **C** Overexpression of *PRKAG2-AS* in AC16 cells was achieved by adenovirus. **D**-**E** Overexpression of *PRKAG2-AS* increased the expression of *PRKAG2b* and *PRKAG2d*. **F**-**G** The expression of *PRKAG2b* and *PRKAG2d* was significantly elevated in DCM heart samples compared to control hearts. **H** There was a positive correlation between the expression of *PRKAG2-AS* and that of *PRKAG2b* and *PRKAG2d* in heart failure. **I** Overexpression of *PRKAG2-AS* increased the expression of atrial natriuretic peptide (*ANP*), brain natriuretic peptide (*BNP*), and β-myosin heavy chain (*Myh7*) while decreasing the expression level of the α-myosin heavy chain (*Myh6*) in AC16 cardiomyocytes. **J** Knockdown of *PRKAG2-AS* in cardiomyocytes decreased the expression of *ANP*, *BNP*, and *Myh7*

Rosiglitazone has been previously reported to increase the risk of worsening heart failure [28, 29]. We wondered whether *PRKAG2-AS* was involved in heart failure by regulating the transcription of *PRKAG2,* and according to the analysis of *PRKAG2-AS* expression in DCM patients, patients with inflammatory dilated heart failure had higher *PRKAG2-AS* in their myocardium (Fig. 7A) (GSE4172 [30]). Additionally, based on our collected heart samples, we found that *PRKAG2-AS* expression was markedly increased in individuals with dilated cardiomyopathy (Fig. 7B). We then used *PRKAG2-AS* adenovirus to overexpress *PRKAG2-AS* in AC16 cells and observed a significant increase in the expression of *PRKAG2b* and *PRKAG2d* (Fig. 7C–E). Furthermore, analysis of heart failure samples showed that the expression of *PRKAG2b* and *PRKAG2d* was significantly higher in patients with dilated cardiomyopathy compared to control hearts (Fig. 7F–G). As evidenced by the positive correlation between *PRKAG2-AS* and *PRKAG2b*/*PRKAG2d* expression in heart failure (Fig. 7H), we concluded that up-regulation of *PRKAG2-AS* may underlie the mechanism of heart failure through regulation of *PRKAG2* transcription in the nucleus.

To investigate the up-regulation of *PRKAG2-AS* in DCM samples, we analyzed the expression of heart failure markers, such as atrial natriuretic peptide (*ANP*), brain natriuretic peptide (*BNP*), β-myosin heavy chain (*Myh7*) and α-myosin heavy chain (*Myh6*), in AC16 cardiomyocytes after both *PRKAG2-AS* overexpression and knockdown. The results showed that overexpression of *PRKAG2-AS* markedly increased the expression of *ANP*, *BNP*, and *Myh7* while reducing the expression level of *Myh6* (Fig. 7I), while knockdown of *PRKAG2-AS* decreased *ANP*, *BNP*, *Myh7* and *Myh6* expression (Fig. 7G). However, we did not observe a substantial change in the expression of heart failure markers upon depletion of *PRKAG2-AS* in the cytoplasm using siRNA (Fig. 7H). These results suggest that up-regulation of *PRKAG2-AS* may play a role in the underlying mechanism of heart failure by regulating *PRKAG2* transcription in the nucleus.

### Elevated expression of *PRKAG2b* and *PRKAG2d* led to abnormal function of hearts

**Fig. 8:**
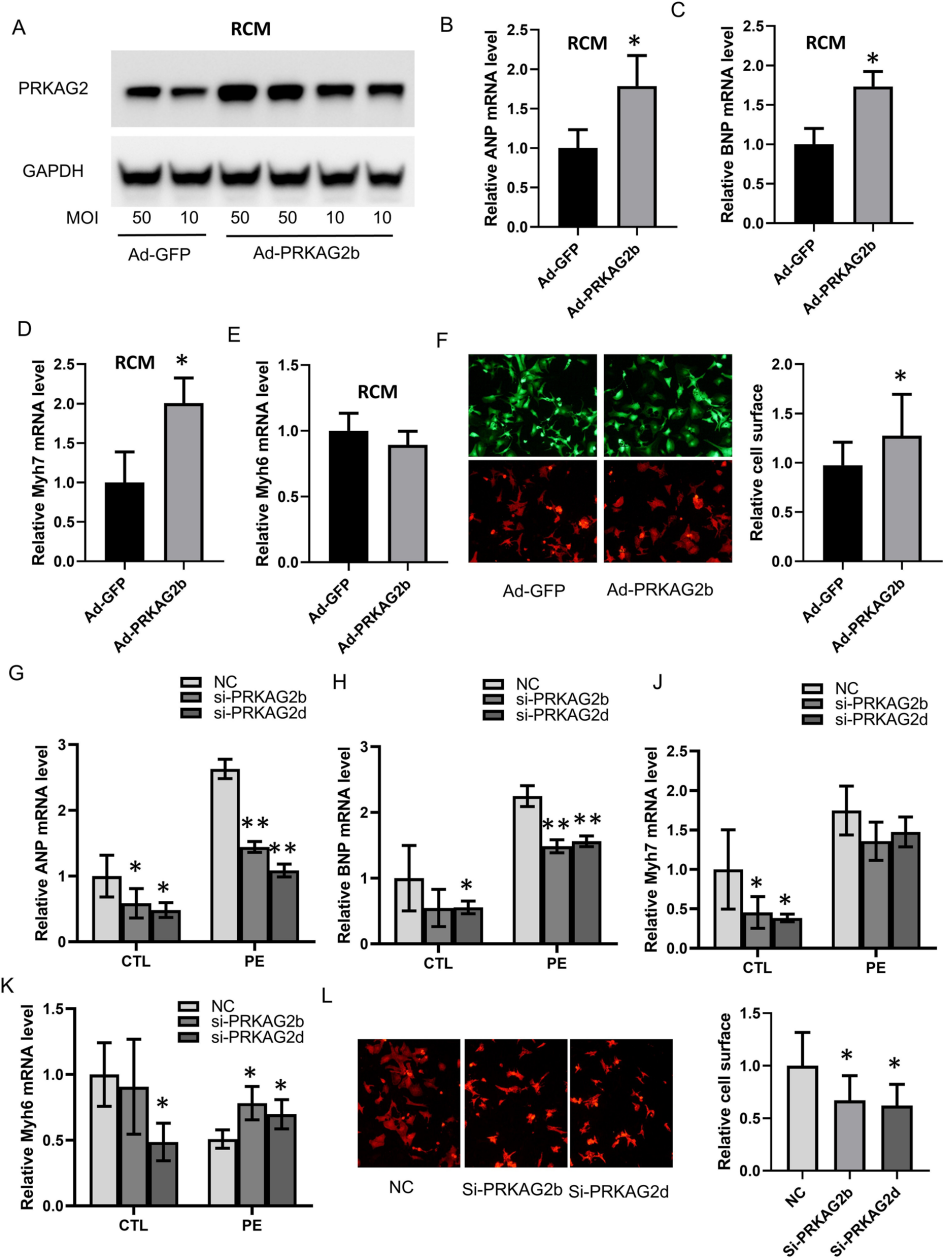
Elevated expression of *PRKAG2b* and *PRKAG2d* leads to abnormal heart function. **A** Western blot confirms elevated *PRKAG2* expression in primary cardiomyocytes transfected with *PRKAG2b* adenovirus at 10 MOI and 50 MOI. **B**–**E** Overexpression of *PRKAG2* increases the expression of *ANP*, *BNP*, and *Myh7*. **F** Overexpression of *PRKAG2* significantly increases cardiomyocyte sizes. **G**–**K** Knockdown of *PRKAG2b* and *PRKAG2d* reduces the expression levels of *ANP*, *BNP*, *Myh7*, and *Myh6* in normal cultured cardiomyocytes and attenuates PE-induced up-regulation of *ANP* and *BNP* in a hypertrophic cardiomyocyte model. (L) Knockdown of *PRKAG2b* and *PRKAG2d* reduces PE-induced enlargement of cardiomyocyte size

To investigate whether the up-regulation of *PRKAG2* in dilated cardiomyopathy (DCM) is involved in heart failure, an adenovirus was constructed to overexpress *PRKAG2b* and transfected into primary cardiomyocytes at 10 multiplicity of infection (MOI) and 50 MOI. Elevated *PRKAG2* expression was confirmed by WB (Fig. 8A). The expression of *ANP*, *BNP*, *Myh7,* and *Myh6* was detected by qRT-PCR, showing that overexpression of *PRKAG2* led to increased expression of *ANP*, *BNP,* and *Myh7* and decreased the expression level of *Myh6* (Fig. 8B-E). Additionally, overexpression of *PRKAG2* significantly increased cardiomyocyte sizes (Fig. 8F). On the other side, knocking down *PRKAG2b* and *PRKAG2d* reduced the expression levels of *ANP*, *BNP*, *Myh7,* and *Myh6* in normal cultured cardiomyocytes and attenuated PE-induced up-regulation of *ANP* and *BNP* in a hypertrophic cardiomyocyte model (Fig. 8G-K). Enlargement of cardiomyocyte size induced by PE treatment was also reduced by *PRKAG2b* and *PRKAG2d* knockdown (Fig. 8L). These results suggest abnormal expression of *PRKAG2b* and *PRKAG2d* might be responsible for the mechanism of *PRKAG2-AS* in heart failure.

### Discussion

**Fig. 9:**
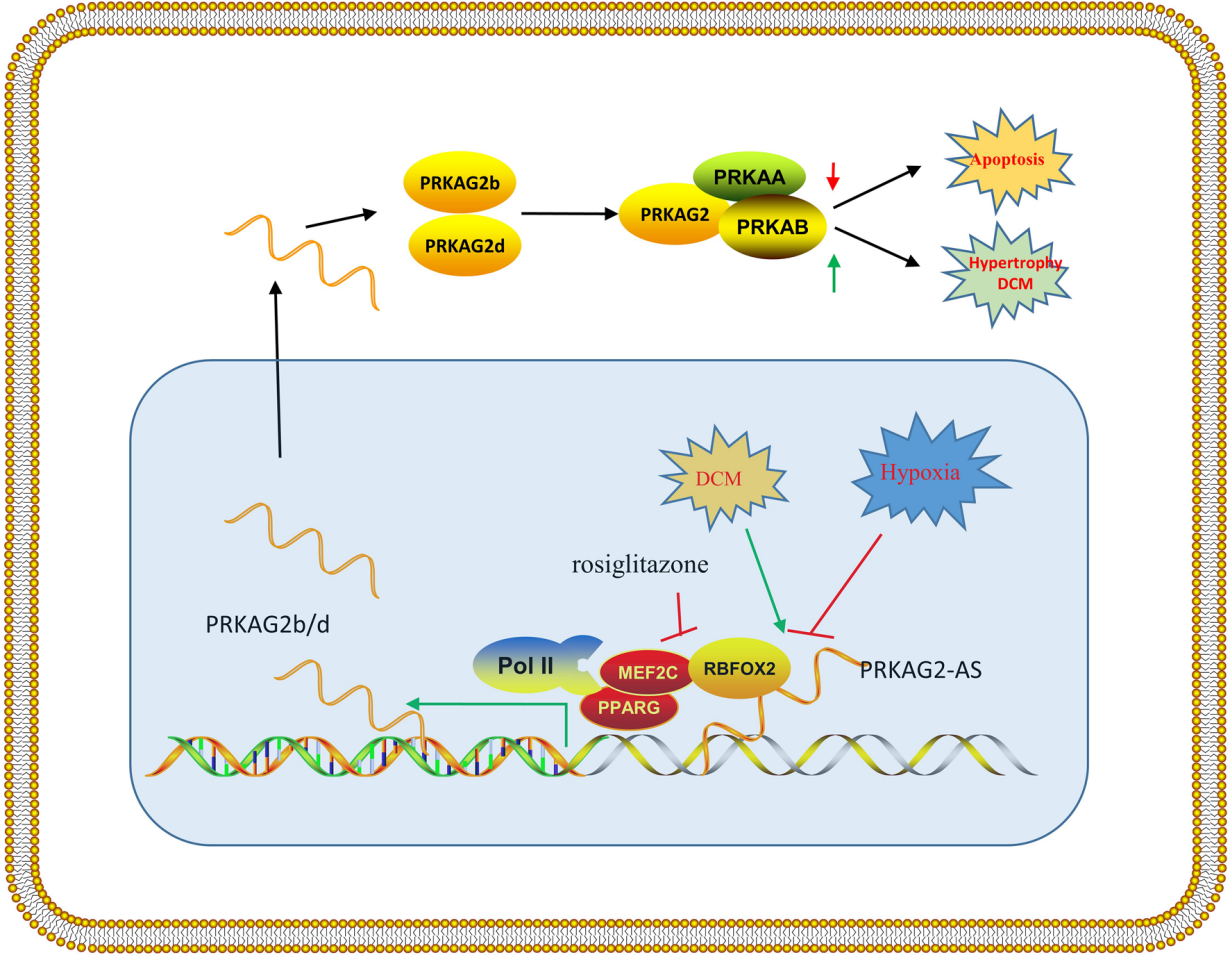
Proper expression of *PRKAG2-AS* is essential for maintaining heart function. *PRKAG2-AS* is down-regulated in cardiac ischemia and up-regulated in DCM. *PRKAG2-AS* interacts with the RBFOX2-PRARG complex to recruit Pol II to the promoter of *PRKAG2*, promoting transcription of *PRKAG2b* or *PRKAG2d* in cardiomyocytes. Aberrant expression of *PRKAG2b* or *PRKAG2d* alters AMPK activity, leading to apoptosis and hypertrophy of cardiomyocytes. Up-regulation of *PRKAG2b* or *PRKAG2d* in heart failure results in abnormal expression of heart failure markers. Therefore, aberrant expression of *PRKAG2-AS* induced by hypoxia or other stressors leads to dysfunction of cardiomyocytes, including death and hypertrophy

*PRKAG2-AS*, a long noncoding RNA, was identified as a crucial regulator of *PRKAG2* expression in cardiomyocytes. As depicted in Fig. 9, we demonstrated the role and mechanism of *PRKAG2-AS* in modulating cardiomyopathy. *PRKAG2-AS* was predominantly expressed in the nucleus. Knockdown of *PRKAG2-AS* in the nucleus led to a significant decrease in the expression of *PRKAG2b* and *PRKAG2d*, highlighting the critical role of *PRKAG2-AS* in maintaining cardiomyocyte function. In the hearts of patients with ischemic cardiomyopathy, *PRKAG2-AS*, *PRKAG2b*, and *PRKAG2d* were all reduced, pointing to a potential involvement of *PRKAG2-AS* in reducing myocardial ischemia. Conversely, overexpression of *PRKAG2-AS* promoted the transcription of *PRKAG2b* and *PRKAG2d*, indicating that up-regulation of *PRKAG2-AS* may contribute to the development of heart failure. Importantly, our results elucidated how PRKAG2-AS regulates PRKAG2 transcription by interacting with factors such as *PPARG*. Accordingly, the study suggests that the proper expression of *PRKAG2-AS* has essential functions in cardiomyocytes, and its aberrant expression induced by hypoxia or other stimuli may result in cardiac dysfunction. Ultimately, our findings may provide new targets for therapeutic intervention in cardiovascular disorders.

We identified the presence of *PRKAG2-AS* on the promoter region of the *PRKAG2* gene using bioinformatics analysis of the human genome. The function and mechanism of *PRKAG2-AS* in cardiovascular disease are not yet fully understood. We assessed the expression of *PRKAG2-AS* in myocardial tissue from healthy individuals and patients with ischemic cardiomyopathy. We found that *PRKAG2-AS* expression was significantly reduced in the ischemic cardiomyopathy group compared to the healthy group. We subsequently conducted siRNA-mediated knockdown of *PRKAG2-AS* in the cytoplasm but did not observe a significant increase in apoptosis of cardiomyocytes. However, we observed a substantial increase in apoptosis when we used antisense oligonucleotides to knock down *PRKAG2-AS* in the nucleus. These findings indicate that knocking down *PRKAG2-AS* expression, particularly in the nucleus, may contribute to ischemia-induced apoptosis in cardiomyocytes. Therefore, the strategies to knock down lncRNAs using siRNAs or antisense oligos depend on the localization of *PRKAG2-AS.* Since *PRKAG2-AS* is mainly found in the nucleus, antisense oligonucleotides may be a more practical approach for *PRKAG2-AS* knockdown. In this manuscript, we only detected the effects of *PRKAG2-AS* and *PRKAG2* on apoptosis by flow cytometry. It is interesting to study the effects of *PRKAG2-AS* and *PRKAG2* on other aspects of the myocardial hypoxia model, such as ultrastructure changes detected by transmission electron microscopy, cell damage-related enzyme activity, and others, in the future.

Based on a review of the literature and the results of our experiments, we hypothesize that elevated expression of *PRKAG2-AS* contributes to heart failure. The expression of heart failure markers such as *ANP* was unaffected by using siRNA to knock down *PRKAG2-AS* in the cytoplasm of cardiomyocytes. However, knocking down *PRKAG2-AS* in the nucleus using antisense oligonucleotides significantly reduced the expression of *Myh6* and *Myh7* while increasing the expression of *BNP* and *ANP*. These findings suggest that *PRKAG2-AS* may modulate heart failure in various ways depending on where it is located inside the cell. *ANP* is secreted by cardiomyocytes and plays a crucial role in regulating natriuretic, vasodilator, and diuretic effects. *BNP*, released following acute myocardial infarction, can reflect the prognosis of patients during the acute phase [20]. Altered expression levels of these hormones are characteristic of cardiac insufficiency. Moreover, *Myh6* and *Myh7* are essential for maintaining cardiomyocyte homeostasis. In future studies, we aim to determine how *PRKAG2-AS* impacts the expression of these molecules and whether its role in heart failure is mediated by their regulation.

We propose that *PRKAG2-AS* may function as a scaffold to recruit transcription factors crucial for controlling the transcriptional of various PRKAG subtypes. In our study, we used two strategies to screen for proteins that might mediate the function of *PRKAG2-AS* in cardiomyocytes, leading to the identification of a representative collection of proteins. It would be fascinating to investigate their mechanisms and contributions to the effects of *PRKAG2-AS* in the context of cardiac ischemia and heart failure. We demonstrated that *PPARG* and PRKAG2-AS are essential in regulating *PRKAG2* transcription. This mechanism may account for the known effects of rosiglitazone on protecting the heart from hypoxia-induced apoptosis of cardiomyocytes and promoting heart failure [29]. Unfortunately, *PRKAG2-AS* is not expressed in rats or mice, which prevents us from directly employing PRKAG2-AS transgenic or knockout rats or mice to study its function in the myocardium. However, another lncRNA located on the promoter region of *PRKAG2* might have a comparable role to *PRKAG2-AS* in the transcriptional regulation of *PRKAG2.*

Previous studies have demonstrated the significant role of *PRKAG2* in heart diseases [31]. We analyzed the expression profiles of five *PRKAG2* subtypes across various tissues, finding that *PRKAG2b* and *PRKAG2d* are predominantly expressed in cardiomyocytes. We observed that decreasing nuclear *PRKAG2-AS* expression significantly reduced the mRNA levels of both *PRKAG2b* and *PRKAG2d*. As *PRKAG2* plays a crucial role in maintaining heart function, these results provide valuable insights into the mechanisms by which *PRKAG2-AS* contributes to heart failure. Our analysis also revealed that *PRKAG2-AS* may directly regulate *PRKAG2* expression in myocardial ischemia. In this condition, the mRNA levels of *PRKAG2b* and *PRKAG2d* were found to be reduced, matching the observed decrease in *PRKAG2-AS* expression. Conversely, in dilated cardiomyopathy (DCM), mRNA levels of both *PRKAG2b* and *PRKAG2d* were elevated and correlated with *PRKAG2-AS* expression. These findings suggest that *PRKAG2-AS* may contribute to the protection of cardiomyocytes from hypoxia-induced apoptosis or abnormal function by regulating the transcription of *PRKAG2b* and *PRKAG2d*. Furthermore, knockdown of *PRKAG2* was demonstrated to affect the expression of heart failure markers consistent with the results of *PRKAG2-AS* knockdown. Moreover, knocking down *PRKAG2b* or *PRKAG2d* could affect cardiomyocyte apoptosis. Collectively, these data indicate that the functionality of *PRKAG2-AS* in heart diseases may be through regulating the expression of *PRKAG2b* and *PRKAG2d*.

Given that *PRKAG2* encodes the regulatory subunit of AMP-activated protein kinase (AMPK) [31], it is intriguing to investigate whether *PRKAG2-AS* is involved in regulating AMPK signaling via the transcriptional regulation of *PRKAG2*. Energy sensing and regulation are activated in cardiovascular diseases, such as myocardial ischemia and stress overload-induced myocardial hypertrophy. Therefore, understanding the function of *PRKAG2-AS* in regulating AMPK activity will help us gain a more comprehensive understanding of cardiovascular diseases and could provide more precise targets for their treatment.
